# NERBio: using selected word conjunctions, term normalization, and global patterns to improve biomedical named entity recognition

**DOI:** 10.1186/1471-2105-7-S5-S11

**Published:** 2006-12-18

**Authors:** Richard Tzong-Han Tsai, Cheng-Lung Sung, Hong-Jie Dai, Hsieh-Chuan Hung, Ting-Yi Sung, Wen-Lian Hsu

**Affiliations:** 1 Institute of Information Science, Academia Sinica, Nankang, Taipei 115, Taiwan, Republic of China

## Abstract

**Background:**

Biomedical named entity recognition (Bio-NER) is a challenging problem because, in general, biomedical named entities of the same category (e.g., proteins and genes) do not follow one standard nomenclature. They have many irregularities and sometimes appear in ambiguous contexts. In recent years, machine-learning (ML) approaches have become increasingly common and now represent the cutting edge of Bio-NER technology. This paper addresses three problems faced by ML-based Bio-NER systems. First, most ML approaches usually employ singleton features that comprise one linguistic property (e.g., the current word is capitalized) and at least one class tag (e.g., *B-protein*, the beginning of a protein name). However, such features may be insufficient in cases where multiple properties must be considered. Adding conjunction features that contain multiple properties can be beneficial, but it would be infeasible to include all conjunction features in an NER model since memory resources are limited and some features are ineffective. To resolve the problem, we use a sequential forward search algorithm to select an effective set of features. Second, variations in the numerical parts of biomedical terms (e.g., "2" in the biomedical term IL2) cause data sparseness and generate many redundant features. In this case, we apply numerical normalization, which solves the problem by replacing all numerals in a term with one representative numeral to help classify named entities. Third, the assignment of NE tags does not depend solely on the target word's closest neighbors, but may depend on words outside the context window (e.g., a context window of five consists of the current word plus two preceding and two subsequent words). We use global patterns generated by the Smith-Waterman local alignment algorithm to identify such structures and modify the results of our ML-based tagger. This is called pattern-based post-processing.

**Results:**

To develop our ML-based Bio-NER system, we employ conditional random fields, which have performed effectively in several well-known tasks, as our underlying ML model. Adding selected conjunction features, applying numerical normalization, and employing pattern-based post-processing improve the F-scores by 1.67%, 1.04%, and 0.57%, respectively. The combined increase of 3.28% yields a total score of 72.98%, which is better than the baseline system that only uses singleton features.

**Conclusion:**

We demonstrate the benefits of using the sequential forward search algorithm to select effective conjunction feature groups. In addition, we show that numerical normalization can effectively reduce the number of redundant and unseen features. Furthermore, the Smith-Waterman local alignment algorithm can help ML-based Bio-NER deal with difficult cases that need longer context windows.

## Background

The exponential growth of large-scale molecular sequence databases and PubMed scientific literature has prompted active research in biological literature mining and information extraction to facilitate genome/proteome annotation and improve the quality of biological databases [1]. Critical tasks in biomedical literature mining include named entity recognition (NER), tokenization, relation extraction, indexing, and categorization/clustering. Using various techniques developed for these tasks, we create a set of tools to assist biomedical researchers in exploiting the stream of publications that are flooding Medline at a rate of 1,500 abstracts a day [2].

Named entity recognition (NER) is a fundamental task that involves the identification of words or phrases that refer to specific entities in texts and their classification into different categories. NER was first defined in the general-language domain in the contests of the Message Understanding Conferences [3]. NER for the biomedical domain (Bio-NER) is a specialized area of NER that has received increased attention in recent years. For natural language processing (NLP) researchers, this field presents a different set of challenges to those found in general NER. In general-language domains, sets of named entities tend to be fairly heterogeneous, ranging from names of individuals to monetary amounts, whereas in the biomedical domain, a set of entities is often restricted to proper biomedical names, such as proteins (e.g., CD28 surface receptor), DNA (e.g., IL-2 gene), RNA (e.g., IL-2 alpha mRNA). Depending on the underlying application, Bio-NER systems can extract objects ranging from protein/gene names to disease/virus names.

Bio-NER presents unique challenges because, in general, biomedical named entities of the same category (e.g., protein, DNA) do not follow one standard nomenclature [4] and can comprise long compound words and short abbreviations [5]. Some NEs contain various symbols and spelling variations [6]. In addition, irregularities often occur in named entities; for example, an NE can have unknown acronyms and contain hyphens, digits, letters, and Greek letters; adjectives preceding an NE may or may not be part of that NE, depending on the context and application; NEs with the same orthographical features may fall into different categories; an NE may belong to multiple categories; and an NE of one category may contain an NE of another category.

Initially, Bio-NER used handcrafted patterns [7] to recognize the various NE forms; however this approach suffered from lack of portability and scalability. Later, machine learning (ML) models were introduced to tackle the Bio-NER problem – first simple classifiers [8,9] and then more complex probabilistic sequence models [10-13]. The first step in an ML approach is to break the input sentence into tokens, usually individual words or hyphenated compounds. Then, each token is assigned a class tag containing its type (e.g., protein, DNA) and position. For example, *B-protein *and I*-protein *are class tags that respectively represent the beginning (*B*) and the internal/ending (*I*) token of a protein name. To make these predictions, ML models rely on linguistic features, i.e., functions that represent linguistic properties and class tags. A linguistic property is a function that indicates the value corresponding to a specific linguistic attribute of the current token, as shown by the following binary function:



Examples of linguistic information that can be referenced in linguistic properties include the affixes of current or neighbor tokens, part-of-speech tags, phrase tags, and specific words. Linguistic features frequently operate within a limited range on either side of the current token, usually two tokens preceding and two following the current token [14]. Such features are categorized as either singleton features or conjunction features [15].

Singleton features are only conditioned on one linguistic property and joined by at least one class tag. For example, a simple binary singleton feature for token-tagging may be "if the current word= 'factor' AND current tag=*I-protein *THEN feature value = 1." Conjunction features, however, are conditioned on multiple linguistic properties and joined by at least one class tag. A conjunction feature similar to the above singleton feature would be "if current word='factor' AND previous word ='transcription' AND current tag=*I-protein *THEN feature value = 1," in which the multiple linguistic properties are current word='factor' and previous word ='transcription'. In most ML systems, singleton features far outnumber conjunction features because the latter occupy a lot of memory. For example, if *n *linguistic properties are used in the ML model, the space required for a conjunction feature is O(*n*^2^) compared to O(*n*) for a singleton feature.

To improve the ML model for Bio-NER, we consider three issues. The first is to choose an effective set of features, both singleton and conjunction, for a given application and hardware configuration. In particular, we want to choose effective conjunction features since they present interesting possibilities [13,16]; however, this is a challenging task.

The second issue we address relates to variations in the numerical parts of biomedical terms (e.g, "2" in the protein name IL2). Such numerical affixes cause data sparseness and generate many redundant features. Furthermore, in the biomedical domain, proteins or genes of the same family may only differ in their numerical parts. For example, interleukin-2 and interleukin-3 belong to the same family – interleukin. In Bio-NER, they are usually annotated as the same NE class. Our solution is to convert all numerals into representative place-holders.

The third issue involves distant tag dependencies in Bio-NER. Most ML models follow the Markov assumption that the current NE tag only depends on the previous tag. However, in Bio-NER, there are many exceptions to this assumption as an NE tag may depend on the previous tag or the next tag, or the words in between. Take "IL-1, IL-2, and AP-1" for example. AP-1's NE class depends on the conjunction "and" and the previous two NEs, so these three NEs should be placed in the same class. However, ML models that operate in a limited context window cannot represent this dependency and are generally weaker at estimating distant features. Furthermore, they may fail if there are dependencies beyond the context window. Therefore, we need a different strategy to model such dependencies.

### Related work

Biomedical NER solution methods fall into three general classes: dictionary-based approaches, rule-based approaches, and machine-learning-based approaches. One might think that systems relying solely on dictionary-based recognition could achieve a satisfactory performance. However, dictionary-based approaches cannot handle unseen NEs and ambiguous contexts effectively [2]. Rule-based approaches, e.g., Fukuda's PROPER system [7], generally rely on combinations of regular expressions (templates) to define patterns that match biomedical NEs and rules for extending NE boundaries to the right and/or left of an expression. For example, a rule-based approach might use a regular expression such as " [a-z]+ [0-9]+ " (a sequence of one or more lower-case letters followed immediately by a sequence of one or more digits) to recognize that p53 is a gene name. One can also create a rule that uses categorical nouns to classify biomedical named entities. For example, compound words ending in "mRNA" have a high probability of being RNA. While rules of this type can be quite effective, they suffer from the weakness of being domain-specific. Thus, if the system is ported to a new domain, many rules would probably need to be modified.

Machine-learning-based approaches are divided into two categories: classifier-based and sequence-model-based. The former include naïve Bayes classifiers and Support Vector Machines (SVM) [8]; and the latter include hidden Markov models (HMM) [11], Maximum Entropy Markov Models (MEMM) [13], and Conditional Random Fields (CRFs) [10]. In the following sections, we discuss the differences between classifier-based and sequence-model-based approaches, and then explain why we choose CRFs for sequence tagging over classifier-based models and other sequence-based models.

#### Formulation

To perform ML-based NER, all sentences must be broken into tokens, which are then given tags. From the numerous token/tag formats available, we adopt the IOB2 format, which is has a proven track record for sequence tagging problems [17]. In IOB2, each word in a sentence is regarded as a token, and each token is associated with a tag that indicates the category of the NE and whether the given token is at the beginning (*B*), or inside (*I*) of the NE. For example, in *B_c*, *I,_c*, where *c *is an NE category, *B_*and, *I *denote, respectively, the first token and the subsequent token of an NE in category *c*. In addition, we use the tag *O *to indicate that a token does not belong to any NE. Once we have tokenized a sentence, we can define NER as the assignment of one of 2*m*+1 tags to each token, where *m *is the number of NE categories. For example, the following phrase annotated in XML format:

"<DNA> IL-2 gene </DNA> expression, <Protein> CD28 </Protein>, and <Protein> NF- kappa B </Protein>"

is transformed to the following IOB2 format:

"IL-2/*B-DNA *gene/I-DNA expression/O,/O CD28/B-protein,/O and/O NF- kappa/B-protein B/I-protein".

#### Classifier-based approaches

In classifier-based approaches, each token is classified into a tag class. For binary classifiers, the training data can be used to train 2*m*+1 classifiers and calculate the token's score for each tag class. For multi-label classifiers, the score for each NE class can be derived directly. After obtaining the score of each tag for each token, the best total tagging sequence for the input sentence using the Viterbi search algorithm [18], which outputs the valid tag sequence with the highest score. In a valid sequence, each *I-*tag must follow either another *I-*tag or a *B-*tag of the same class. For example, *I*-*protein *should follow *I*-*protein *or *B*-*protein*. An invalid tag sequence would be one containing a *B*-*protein *followed by an *I*-*DNA*.

#### Sequence-based models

Sequence-based models can use the same token/tag format as classifier-based models. However, in sequence models, features must refer to tags in the context window. In most cases, they refer to the current, preceding or subsequent tags. We can divide sequence models into generative and discriminative types.

#### HMM and MEMM

The Hidden Markov Model (HMM) is a generative type of sequence-based model. In the following explanation of sequence models, **x **refers to the input token sequence and y refers to the output tag sequence. Generative models generally find the best tag sequence by computing the generative form of the probability *p*(**x**,**y**). In HMM, the probability of *p*(**y**|**x**) can be rewritten as a calculation utilizing its generative form *p*(**x**,**y**) according to the Bayes theorem:



Assuming the current tag *y*_*i *_depends on the previous tag *y*_*i*-1_, and the current token *x*_*i *_depends on the current tag *y*_*i*_, then *p*(**x,y**) can be rewritten as:



where *n *is the number of tokens in **x**.

Since the objective is to find the best *p*(**y**|**x**), and *p*(**x**) is an a priori probability that remains the same for each possible tag class, we only need to compare *p*(**x**,**y**).

The problem with Equation (2) lies in data sparseness caused by *p*(*x*_*i*_*|y*_*i*_). Ideally, we would have sufficient training data for every possible value of *x*_*i *_in order to calculation of *p*(*x*_*i*_*|y*_*i*_). In reality, however, there is rarely enough training data to compute accurate probabilities when decoding new data. This problem is often solved by using the naïve Bayes approach.

In the the naïve Bayes approach, we decompose *p*(*x*_*i*_*|y*_*i*_) as follows:



where *f*_*ij *_is the value of *x*_*i*_'s *j*^th ^feature.

Even with the above solution, HMMs suffer from two limitations. The first arises from the naïve Bayes assumption of HMMs for solving NER, which would benefit from a richer representation of observations, especially a representation that describes observations in terms of many overlapping features, such as capitalization, affixes, part-of-speech (POS) tags, in addition to surface word features. For example, when trying to extract unseen company names from a newswire article, knowing whether the word is capitalized and associated with a POS noun tag would be useful. However, a naïve Bayes assumption might fail in this case because these features are not independent of each other. The second problem with HMM is that it sets its parameters to maximize the likelihood of the observation sequence, but the task is to predict the state sequence given the observation sequence. In other words, HMM inappropriately uses a generative joint model to solve a conditional problem in which the observations are given.

Maximum entropy Markov models (MEMMs) [19] have been proposed to address both of the above issues. To allow for non-independent observation features that are difficult to enumerate, MEMM replaces the generative, joint probability parameterization employed by HMMs with a conditional model that represents the probability of reaching a state given an observation feature and the previous state. The probability of *p*(**y|x**) in MEMM is calculated as follows:







where *Z*(*y*_*i*-1_, **x**) is the normalization factor that ensures the probability of all state *y*_*i *_sum to one, *h*_*j*_(*y*_*i*-1_, *y*_*i*_, **x**, *c*) is usually a binary-valued feature function and *λ*_*j *_is its weight. Large positive *λ*_*j *_values indicate a preference for such an event, whereas large negative values make the event unlikely.

However, despite their advantages, MEMMs suffer a label bias problem in that the transitions leaving a given state only compete against each other, rather than against all other transitions in the model [20,21]. The Markovian assumptions in MEMMs ignore the dependencies between the current state and other states, except for the previous state. To resolve this problem, Lafferty et al. [21] developed conditional random fields (CRFs), a sequence modeling framework that retains the advantages of MEMMs, but avoids the label bias problem.

#### Conditional Random Fields

CRFs are undirected graphical models, in which each node represents a state that is trained to maximize a conditional probability [21]. A linear-chain CRF with parameters Λ = {*λ*_1_, *λ*_2_, ...} defines a conditional probability for a state sequence **y **= *y*_1 _...*y*_*n *_given a length-*n *input sequence **x **= *x*_1 _...*x*_*n *_as follows:





where *Z*(**x**) is the normalization factor that ensures the probability of all state sequences sum to one, *C *is the set of all cliques in the target sentence, and *c *is any single clique. A clique is a fully connected subset of nodes. Note that *h*_*j*_(**y**_*c*_, **x**, *c*) is usually a binary-valued feature function and *λ*_*j *_is its weight. Large positive *λ*_*j *_values indicate a preference for such an event, while large negative values make the event unlikely.

The size of *c *determines which states *h*_*j *_can refer to. If *c *contains only the current state, then *h*_*j *_can only refer to the current state. However, if *c *contains the current and the previous states, then *h*_*j *_can refer to all of them. Given **x**, the conditional probability of **y **is equal to the exponential sum of *λ*_*j*_*h*_*j *_in all cliques. Since the average length of a biomedical NE is between two and three tokens, we use two instead of three for the clique size to economize on memory space. The following are two examples of current word features. The first refers to the pair *y*_*i *_and *y*_*i*-1 _and the second refers to *y*_*i *_individually:



where **y**_*c *_stands for the tag sequence in *c*, and [*i*-1, *i*] means that the clique *c *ranges from the *i*-1^th ^token to the *i*^th ^token.

**Figure 1:**
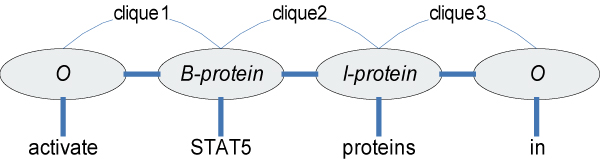
Graphical representation of "activate STAT5 proteins in" tagged as "[*O, B-protein, I-protein, O*]".

Figure 1 shows a graphical representation of "activate STAT5 proteins in" tagged as " [*O*, *B-protein*, *I-protein*, *O*]". To calculate the probability of the phrase "activate STAT5 proteins in" being tagged as [*O*, *B-protein*, *I-protein*, *O*], we consider the three cliques involved: [activate, STAT5], [STAT5, proteins], and [proteins in], as shown in Figure 1. Then, given the input sequence, the conditional probability of the specified tag sequence can be calculated as follows:



The most probable label sequence for **x**,



can be efficiently determined using the Viterbi algorithm [18].

The parameters can be estimated by maximizing the conditional probability of a set of label sequences, given each of their corresponding input sequences. The log-likelihood of a training set {(**x**_*l*_, **y**_*l*_): *l *= 1, ..., *M*} is written as:



To optimize the parameters in CRFs, we use a quasi-Newton gradient-climber BFGS [22].

#### Comparison of HMM, MEMM, and CRF

The critical difference between CRF and MEMM is that the latter uses per-state exponential models for the conditional probabilities of next states given the current state, whereas CRF uses a single exponential model to determine the joint probability of the entire sequence of labels, given the observation sequence. Therefore, in CRF, the weights of different features in different states compete against each other.

After comparing the various models, we chose CRF as our framework. Its primary advantage over HMM is its conditional nature, which allows for the relaxation of the independence assumptions that HMM requires to ensure tractable inferences. Additionally, CRF avoids the label bias problem [21] inherent in MEMM [19] and other conditional Markov models based on directed graphical models [23]. CRF outperforms both MEMM and HMM in a number of real-world sequence labelling tasks [21,24,25]. In addition, CRF uses exponential weighed sums to combine the influences of many correlated features, including overlapping and co-dependent features. As a result, we can use multiple features with CRF more easily than with HMM.

## Results and Discussion

### Datasets

In our experiment, we employ the dataset used in the JNLPBA 2004 shared task [26], which was converted from the GENIA corpus. The GENIA corpus was formed by a controlled search of MEDLINE using the MeSH terms "human", "blood cells" and "transcription factors". In that search, 2,000 abstracts were selected and annotated manually according to a taxonomy of 48 classes, of which 36 were used for annotation. Several biomedical NER systems use the GENIA corpus as training and test data [9,27].

In the JNLPBA 2004 shared task, the GENIA corpus was still used as training data. However, the original 36 classes were simplified to 5 classes: protein, DNA, RNA, cell line, and cell type. To reduce the annotation task to a simple linear sequential analysis problem, embedded structures have been removed leaving only the outermost structures. Consequently, a group of coordinated entities involving ellipsis are annotated as one structure, as shown by the following example:

... in [lymphocytes] and [T- and B-lymphocyte] count in ...

In the example, the term "T- and B-lymphocyte" is annotated as one structure even though it involves two entity names, "T-lymphocyte" and "B-lymphocyte", whereas "lymphocytes" is annotated as one entity.

To ensure that the evaluation was objective, the JNLPBA task organizers provided 404 newly-annotated MEDLINE abstracts from the GENIA project as test data. They were annotated with the same five entity categories as the training dataset. Half of the abstracts were from the same domain as the training data and the other half were from the super-domain of "blood cells" and "transcription factors". Here, a domain refers to a specific subject or area of knowledge, while a super domain is a broader subject area than a domain. The super-domain of "blood cells" and "transcription factors", for example, includes more general terms, such as cells and proteins. Testing on the super-domain can provide an important measure of the generality of the methods used. We also used this dataset as the test set. The basic statistics for the training and test data are summarized in Table 1.

### Evaluation methodology

Results are reported as F-scores using JNLPBA's evaluation script, which is a modified version of the evaluation script of the CoNLL-03 shared task [28]. The F-score is defined as F = (2PR)/(P + R), where P denotes the precision and R denotes the recall, defined as follows:



The evaluation script outputs three sets of F-scores according to the exact boundary matching, right-boundary matching, and left-boundary matching [29]. In the right-boundary matching, we only examine whether the right boundaries of entities match the true NEs, without considering the left boundaries. Left-boundary matching is performed in a similar manner.

### Feature denotation

Before describing the system and experimental results in depth, we explain how features are denoted. We group feature functions by their linguistic properties, denoted by italicized letters and subscript appearing at the left-hand side of the equation phrase. For example, the feature functions that refer to the same linguistic property *w*_*i*_, such as "*w*_*i *_= IL1", "*w*_*i *_= IL2", and "*w*_*i *_= IL3" are grouped as the feature group "current word". Feature groups can also be combined into feature types. For example, the current word, previous word, and next word (*w*_*i*_, *w*_*i*-*1*_, and *w*_*i*+*1*_) form a "word" feature type.

Our system uses six singleton feature types: word, orthographical features, part-of-speech (POS), word shape, affix, and chunk. They are described in the Methods section along with some of their conjunctions.

## Results

Table 2 shows the improvements in NER performance achieved by incrementally adding new features to the baseline model, which defines our three methods. The model is also described in the Methods section. In the table, F_singleton _denotes the feature set of all six singleton feature types, and F_conjunction _refers to the set of the selected word conjunction feature groups. NN indicates that numerical normalization was applied to both the training and the test data, while PBPP indicates that pattern-based post-processing was applied to the results of NN. The #1 configuration, which simply employs F_singleton, _(Note comma) is our baseline configuration. The configurations created by adding F_conjunction, _NN, and PBPP to F_singleton _one-by-one are denoted as #2, #3, and #4, respectively. We observe that the F-scores increase by 1.67%, 1.04%, and 0.57%, respectively. For a Bio-NER system with an F-score of over 70%, these improvements are appreciable. Configuration #4 is our system for Bio-NER, called NERBio.

In Table 3, we list the precision, recall, and F-score for each category of NE. We observe that the F-scores for proteins and cell types are comparatively high. This is possibly because they comprise two of the top three most frequent categories in the training set (as shown in Table 1). However, although DNA is the second most frequent category, it does not have a high F-score. We think this discrepancy is due to the fact that DNA names are commonly used in proteins, causing a substantial overlap between these two categories. RNA's performance is comparatively low because its training set is much smaller than other categories. The performance on cell line is the lowest since it overlaps heavily with cell type and its training set is also very small. In Table 4, we compare NERBio with the top three JNLPBA 2004 systems. NERBio performs better than [13] and [10], which use MEMM and CRF, respectively, because our conjunction features, term normalization, and pattern-based post processing are effective.

Surprisingly, Zhou's HMM [12] outperforms Settles' CRF model [10], which seems to contradict our earlier comments. Settles' model simply uses general, non-biomedical features, such as word and orthographic features, whereas Zhou's model incorporates domain-specific resources, such as biomedical dictionaries, manually observed rules, abbreviations, and an alias database. These additional resources probably fill in gaps when the system encounters unseen words.

Table 5 shows the F-scores for the following boundary matching criteria: exact boundary match (Exact Match), left boundary match (Left Match) and right boundary match (Right Match). By relaxing the boundary matching, the F-score improves from 3.11% (Left Match) to 6.56% (Right Match). Although this increases the tagging accuracy of some NEs that have descriptive preceding adjectives or rightmost head nouns, it also increases other types of errors. The degree of relaxation should be based on the specific NER application [29].

## Discussion

Recognition disagreement between our system results and the JNLPBA corpus can be attributed to the following two factors:

### Annotation problems in the JNLPBA Corpus

Although inter-annotator agreement results for the JNLPBA corpus are not available, some studies of inter-annotator agreement among biomedical named entities have reported agreement rates between 87% [30] and 89% [31]. We divide the annotation problems into four sub-problems, all of which are caused by inconsistent annotation.

#### (a) Preceding adjective problem

Some descriptive adjectives are annotated as part of the subsequent NE, but some are not. In fact, it is even hard for biologists to decide whether descriptive adjectives, such as "normal" and "activated", should be part of entity names. For example, in the training data, "human" occurred 1,790 times either before or at the beginning of an entity, but it was not recognized as a part of an entity 110 times. In test data, however, it was only excluded from an NE once out of 130 occurrences. This irregularity confuses NER systems and reduces the reliability of evaluation results on the GENIA corpus.

#### (b) Nested NE problem

In the JNLPBA data, we found that, in some instances, only embedded NEs are annotated, but in other instances, only the outmost NEs are annotated. In fact, both should be tagged. However, according to the JNLPBA's simplification of NER, which removes all embedded NEs, the outmost NE should be tagged. For example, in the training set of the JNLPBA 2004 data, in 59 instances of the phrase "IL-2 gene", "IL-2" was annotated as a protein 13 times, while in the remaining 46 instances "IL-2 gene" was tagged as DNA. This irregularity can confuse machine learning based systems.

#### (c) Cell-line/cell-type confusion

NEs in the cell line class are from certain cell types. For example, the HeLa cell line can be from humans or cellular products. Given the abbreviated content of an abstract, it is even difficult for an expert to distinguish between cell lines and cell types. In GENIA, most instances of "granulocytic colonies" are tagged as cell lines; however, in the phrase "stimulated primary murine bone marrow cells to form granulocytic colonies in vitro", the phrase "granulocytic colonies" is tagged as a cell type.

#### (d) Missing tags

In the training data of the JNLPBA 2004 data, some NEs of each category, especially cell lines, are not tagged. Such incorrect annotation causes a large number of false negatives. For example, we have observed many instances of "T cell", "Peripheral blood neutrophil", and "NK cell" not tagged as cell lines.

### System recognition errors

The other main cause of disagreement is our system's tagging errors. We categorize errors into two subtypes:

#### (a) Misclassification

Some protein molecules or regions are misclassified as DNA molecules or regions. These errors may be solved by exploiting more context information.

#### (b) False positives

Some entities appear without a specific name, e.g., "the epitopes" without indicating which kind of epitopes. GENIA tends to ignore these entities, but their contexts are similar to the entities with specific names. Therefore, our system sometimes incorrectly recognizes them as NEs.

## Conclusion

In the biomedical domain, relying solely on singleton features cannot resolve all ambiguous cases. Adding conjunction features is necessary, therefore, and has proven effective previously [13]. However, any conjunction feature group, especially the word conjunction feature group, has many more member features than any singleton feature group. It quickly becomes infeasible to include a large number of conjunction feature groups in a Bio-NER ML model due to limited memory resources. Furthermore, including all feature groups may not be desirable since features in some feature groups occur rarely and therefore have inaccurate weights. In this paper, we successfully employ sequential forward search (described in the Methods section) to select the most effective feature groups. The next problem we address is data sparseness caused by variations in the numerical parts of Bio-NEs. Such variations generate many redundant and unseen features. To deal with these variations we apply numerical normalization. Lastly, we overcome the limits of the context window using pattern-based post-processing. A token's tag may not only depend on its adjoining neighbors, but may be determined by words beyond the context window. We use automatically generated global patterns to recognize distant relations and modify the CRF tagging results accordingly. Using the above three methods, we improve the system's overall performance by 3.28%, achieving a total F-score of 72.98%, which is higher than all current JNLPBA systems.

There are still several unsolved problems in Bio-NER. For example, it is still difficult to recognize long, complicated NEs and to distinguish between two overlapping NE classes, such as cell-lines and cell-types. Another serious problem is annotation inconsistency, which confuses machine learning models and makes evaluation difficult.

Certain errors, such as those in boundary identification, are relatively tolerable if the main purpose is to discover the relations between NEs. In our future work, we will exploit more global linguistic features, e.g., semantic role labels and full-parsing features. To date, we have not exploited external databases, such as iProLink [1]; however, we will incorporate them into our system. Finally, to reduce the requirement for human annotation and alleviate the scarcity of available annotated corpora, we will develop machine learning techniques to apply to partially annotated corpora in different biomedical domains.

## Methods

In this section we describe numerical normalization, the feature set of our CRF model and its selection, as well as global pattern-based correction post-processing.

### Feature set

Feature selection is critical to the success of machine learning approaches. In this section, we describe the features used in our system.

#### Word features

Words preceding or following the target word may be useful for determining its category. Take the sentence "The IL-2 gene localizes to bands BC on mouse Chromosome 3" for example. If the target word is "IL-2", the following word "gene" will help the CRF model distinguish the IL-2 gene from the protein of the same name. One might assume that the larger the context window, the better and more precise the results will be. However, widening the context window would lead to an explosion in the number of word features, often encompassing infrequent word features that are distant from the current token. In our experience, a suitable window size is five, i.e., the two preceding words, the current word, and the two following words. This window size is also suitable for most tagging problems, such as POS tagging [32].

#### Orthographical features

Table 6 lists all the orthographical features used in our system. These features are widely used in other general NER [33] or biomedical NER systems [12]. Empirically it has been shown that these features can detect NE patterns. Take the ALPHANUMERIC feature for example. The digits following a sequence of English letters, e.g., '5' in the protein STAT5, usually denote the serial number of the gene, protein, or cell families. These digits can be used to distinguish Bio-NEs from general English words.

#### Part-of-speech features

Part-of-speech (POS) information is useful for identifying named entities. Verbs and prepositions usually indicate an NE's boundaries. Nouns not found in the dictionary are usually proper nouns, which are good candidates for named entities. Setting a context window length for POS features is similar to setting the window length for word features. We found that five is also a suitable window size for POS features. The Stanford POS tagger [34] is used to provide POS information. We trained it on GENIA 3.02 p and achieved 98.85% accuracy.

#### Word shape features

Sometimes, named entities belonging to the same category are similar, for example, HLA-A and HLA-B. We first employ a simple method to normalize all similar words: all capitalized characters are all replaced by 'A'; all digits are all replaced by '0'; non-English characters are replaced by '_' (underscore); and non-capitalized characters are replaced by 'a'. Thus, Kappa-B would be normalized as "Aaaaa_A". To further normalize such words we reduce consecutive strings of identical characters to a single character. This feature is the *compressed word shape feature*. For example, "Aaaaa_A" is normalized to "Aa_A". After applying the first normalization method, the two proteins "HLA-A" and "HLA-B" will normalized to the same term "AAA_A" and activate the same features. After applying the second method, "IL-2" and "IL-21" will be normalized to the same term "A_1" and activate the same features.

#### Affix features

An affix is a morpheme that is attached to a base morpheme, such as a root or a stem, to form a word. The type of an affix depends on its position relative to the root. Prefixes (attached before another morpheme) and suffixes (attached after another morpheme) are two of these types. Some prefixes and suffixes provide good clues for classifying named entities. For example, words that end in "~ase" are usually proteins. However, short prefixes or suffixes are too common to be of any help in classification. For example, it would be difficult to guess to which category a word ending in "~es" belongs. In our experience, the acceptable length for prefixes and suffixes is 3–5 characters; the longer the prefix or suffix, the fewer the number of matches that will be found.

#### Chunk features

Chunk features, which are provided by a chunker or shallow parser, are also useful for recognizing NEs. In shallow parsing, a sentence is divided into a series of chunks that include nouns, verbs, and prepositional phrases. Generally speaking, NEs are usually located in noun phrases. In most cases, either the left or right boundary of an NE is aligned with either edge of a noun phrase. For instance, in the noun phrase "the human interleukin-2 gene", the gene name "human interleukin-2 gene" aligns with the right boundary of the noun phrase. NEs rarely exceed phrase boundaries. Our system uses the GENIA tagger [35] to obtain chunk data.

#### Conjunction features

As the above features are all singletons, in some cases, they are insufficient to classify tokens correctly. Consider the following two features:



Neither feature is effective by itself. When the first feature is enabled, the current tag can be either *I-protein*, *I-DNA*, or *O*, depending on whether the current word is "factor," "silencer ", or "rate". However, their conjunction



is very effective because when the previous word is "transcription," and the current word is "factor," the current word is most likely the last word of a transcription factor name, which is categorized as a protein name in the GENIA ontology.

### Feature group selection

Each feature type has several feature groups that differ in their positions, lengths, and patterns. Since not all feature groups are effective for Bio-NER, we need to select effective feature groups. Performing feature group selection on all possible groups is extremely time-consuming. Therefore, following previous NER studies, we only consider lengths ranging from 3 to 5 characters, and positions ranging from -3 to +3 tokens relative to the current token. Table 7 lists the feature types, the number of subtypes and the number of possible feature groups in each feature type. We can see that both the word and POS feature types have seven feature groups, which differ in their relative positions. Some feature types have subtypes, such as the orthographical feature type, which has 17 subtypes, giving it 7 × 17 = 119 feature groups. The word shape feature type has two subtypes, word shape and compressed word shape; while the affix feature has two subtypes, prefix and suffix. The word conjunction feature has  feature groups. The total number of all potential feature groups is 210.

Because our system has tens of thousands of singleton features, employing all possible conjunctions is infeasible. In our experience, one word conjunction feature group (e.g., *w*_*i*-1_=*X*_1 _AND *w*_0_=*X*_2_, where *X*_1 _and *X*_2 _can be any word) occupies approximately 1.5 GB of RAM. Therefore, a server with 10 GB of RAM can only accommodate about six word conjunction feature groups. Even though including all the word conjunction features is computationally feasible, it would be difficult to obtain sufficient statistics for these features since most conjunctions occur rarely, if ever. Therefore, the performance would be reduced. In [15], it is reported that the performance achieved using all singleton features and all conjunction features is not as good as that derived by only using the best subset in the CoNLL-2003 English NER task's dataset. Given the limited memory, we need to use a suitable feature-selection algorithm to maximize the system's performance.

Feature selection is often viewed as a search problem in a space of feature subsets. To carry out this search we must specify a starting point, a strategy to traverse the space of subsets, an evaluation function, and a stopping criterion. Although this formulation allows a variety of solutions to be developed, usually two families of methods are considered: filter and wrapper. Filter methods use an evaluation function that relies solely on the properties of the data, making them independent of any particular machine learning algorithm. Commonly used measurements include mutual information and information gain. In the field of CRF-based sequence tagging, [15] describes an implementation of feature induction for CRF that automatically creates a set of useful features and conjunction features. The method scores candidate features by their log-likelihood gains. Feature induction works by iteratively considering sets of candidate singleton and conjunction features created from the initially defined set of singleton features as well as the set of current model features. Only candidates with the highest gain are included into the current set of model features. Intuitively, features with high gain provide strong evidence for many decisions. To calculate the log-likelihood gain efficiently, McCallum [15] makes certain independence assumptions about the parameters and only includes positions in the sequence that are mislabeled by the current parameter settings. In biomedical NER, McDonald et al. [16] applied McCallum's method and achieved a 2% increase in the F-score.

Wrapper methods use the actual evaluation to measure the quality of *F*, where *F *is a subset of all feature candidates. In this approach, a small portion of data is selected for training and development initially. The evaluation is then performed on the development set. Several machine learning and pattern recognition papers [36-38] have established that the wrapper method can select more appropriate features for a specific machine learning algorithm than the filter method. In the current work, we also adopted the wrapper method.

Due to time limitations, it is very difficult to select a globally optimal feature set for the development set. In our system, we employ sequential forward selection to find the best feature subset. We first calculate which feature group has the highest F-score and select it as the basis for the feature pool. In each subsequent iteration, we add the feature groups to the feature pool individually and calculate their F-scores. Each time, we select the feature group with the best score and add it to the pool. This process continues until the F-score stops increasing.

Currently, we only include word conjunction features because they are more effective than other conjunctions. Table 8 shows the most effective word conjunction feature groups selected by our algorithm. These features specify the values of two words in the context window. For example, *w*_*i*-1 _AND *w*_*i *_stands for all features that contain the two properties "*w*_*i*-1_=* " and "*w*_*i*_=*", where '* ' can be any word. The performance of the selected conjunction features is discussed in the Results section.

### Numerical normalization

Numerical normalization is a data preprocessing method that converts numerals in each term to one representative numeral. The advantages of numerical normalization include: (1) the number of features can be substantially reduced; (2) it is possible to transform unseen features into seen features; and (3) feature weights can be estimated more accurately. Take the gene names IL2, IL3, IL4, and IL5 for example. IL2, IL3, IL4 are in the training set, but IL5 is not. If we apply numerical normalization to these terms, they will all be normalized to IL1. Therefore, the number of features corresponding to the first three terms is reduced to a minimum of 1/3. Since IL5 and IL1 are treated alike and share the same weight, this unseen feature becomes a seen feature. According to our results, normalization generally increases overall Bio-NER accuracy (Table 2). Suppose IL2 is annotated as "gene" three times, IL3 is annotated as "gene" six times, and IL4 is annotated as "gene" once and as "compound" once. The annotation of IL4 may confuse machine learning models. After numerical normalization, however, the first three terms are annotated as "gene" ten times and as "compound" only once. Therefore, the feature weights can be correctly estimated.

### Using global pattern to improve CRF

Since dependency may exist between NEs, words among NEs, and even words beyond the context window (as described in Background section), we apply global patterns composed of NEs and surrounding words to resolve the problem. In the following subsections, we describe pattern induction, pattern filtering, and pattern-based error correction in detail.

#### Global pattern induction and filtering

The first step of creating global patterns applies numerical normalization to all sentences in the training, development, and test sets. For each pair of sentences in the training set, we apply the Smith-Waterman local alignment algorithm [39] to find the longest common string, which is then added to the candidate pattern pool. During the alignment process, positions where the two input sequences share the same word or NE class are counted as a match. Here, NE class means a tag's NE category, such as *cell_type, protein, RNA*, or *DNA*. The similarity function used in the Smith-Waterman algorithm is:



where *x *and *y *refer to any two compared tokens from the first and second input sentences, respectively. The similarity of two inputs is calculated by the Smith-Waterman algorithm based on this token-level similarity function.

The following two tagged sentences show how patterns are extracted from a sentence pair in the training set:

"**both**/*O *megakaryocytic/***B-cell_type ***and/***I-cell_type ***erythroid/***I-cell_type ***lineages/**I-cell_type**/*O*" and

"**both**/*O *myeloid/***B-cell_type ***and/***I-cell_type ***natural/***I-cell_type ***killer/***I-cell_type ***(/***I-cell_type ***NK/***I-cell_type***)/***I-cell_type ***cells/***I-cell_type***/O"

We generate a pattern "**both <cell_type>**." for them. Here, we put the aligned words and tags in bold font. The first and last tokens in a pattern are constrained to be words, or sentence beginning and ending symbols.

The extracted patterns are composed of a headword, an NE type, and a tail-word; for example, "headword <NE type> tail-word". To test the patterns' effectiveness, each one is applied to the development set to correct the NE tags of all sentences. If the pattern's error ratio exceeds a certain threshold, τ, the pattern is filtered out.

#### Complexity analysis

For each pair of sentences in the training set (*n *sentences), using local alignment to find their longest common patterns has a complexity of *O*(*n*^2^*l*^2^), where *l *is the longest sentence in the training set. The evaluation of each pattern, *p*, using the development set (*m *sentences) has a complexity of O(*mnl*^2^).

#### Error correction

After the CRF-based NER module tags an input sentence, we check if that sentence can be corrected by our global patterns. We first modify the tagged sentence for matching. The tagged sentence output by CRF is still in the IOB2 format. For the tokens assigned to an NE, we combine them with an NE-type symbol, while for others, we only keep the words. For example, "both/*O *CD4/*O *and/*O *CD8/*B-cell_type *mature/*I-cell_type *T/*I-cell_type *cells/*I-cell_type*/*O*" is modified to "both CD4 and <cell_type>.".

Next, we match the transformed tagged sentence *s*' with any pattern *t*. Basically, the matching is also implemented using the Smith-Waterman local alignment algorithm. After this matching, two aligned segments have the same beginning and end tokens (words or symbols). For each aligned pair of segments *p *in *s*' and *q *in *t*, we check if both *p *and *q *contain the same NE symbol *σ*. If they do, we check the gap ratio *γ *as follows:



Our correction policy is very simple. If *γ *is less than a threshold ζ, we modify *p *to be *σ*, except for beginning and end words.

Let us use the above example to explain pattern matching and error correction. We align the modified segment "both CD4 and <cell_type>" with a pattern segment "both <cell_type>.". There are two gaps. Therefore, *γ *is equal to 2/(8-2) = 0.33, which is less than our threshold of 0.4. Then, we modify the original tagged segment to be "both/*O *CD4/***B_cell_type ***and/***I-cell_type ***CD8/***I-cell_type ***mature/*I-cell_type *T/*I-cell_type *cells/*I-cell_type*/*O*", where the modified tags are in bold font.

## Authors' contributions

RTH Tsai designed all the experiments. CL Sung developed and implemented the pattern-based post-processing algorithm. HJ Dai implemented the sequential feature selection algorithm. RTH Tsai and HC Hung implemented the numerical normalization subroutine and conducted all experiments. TY Sung and WL Hsu guided the whole project.

## Figures and Tables

**Table 1:** Absolute (and relative) frequencies for NEs in each data set

	protein	DNA	RNA	cell type	cell line	All
Training Set	30,269	9,533	951	6,713	3,830	51,301
	(15.1)	(4.8)	(0.5)	(3.4)	(1.9)	(25.7)
Test Set	5,067	1,056	118	1,921	500	8,662
	(12.5)	(2.6)	(0.3)	(4.8)	(1.2)	(21.4)

**Table 2:** NER performance of each configuration on the JNLPBA 2004 data

	F_singleton_	F_conjunction_	NN	PBPP	P(%)	R(%)	F(%)
1	√				68.60	70.90	69.70
2	√	√			70.31	72.47	71.37
3	√	√	√		71.43	73.41	72.41
4	√	√	√	√	72.01	73.98	72.98

F_singleton, _F_conjunction,_NN, and PBPP denote the baseline features, conjunction features, numerical normalization, and pattern-based post-processing, respectively.

**Table 3:** NER performance of each NE category on the JNLPBA 2004 data

NE category	Precision (%)	Recall (%)	F-score (%)
Protein	71.31	79.36	75.12
DNA	71.70	68.37	70.00
RNA	70.08	75.42	72.65
cell line	56.24	58.60	57.39
cell type	79.94	66.79	72.77
Overall	72.01	73.98	72.98

**Table 4:** NER performance comparison of top systems on the JNLPBA 2004 data

System	Model	Precision (%)	Recall (%)	F-score (%)
NERBio	CRF	72.01	73.98	72.98
[12]	HMM	69.40	75.98	72.55
[13]	MEMM	68.60	71.60	70.10
[10]	CRF	69.30	70.30	69.80

**Table 5:** F-score of each NE category for different matching criteria

NE category	Exact Match (%)	Left Match (%)	Right Match (%)
Protein	75.12	79.15	80.91
DNA	70.00	71.64	76.49
RNA	72.65	74.29	77.55
cell line	57.39	60.33	66.41
cell type	72.77	74.08	81.11
Overall	72.98	76.09	79.54

**Table 6:** Orthographical features

Feature name	Regular Expression
INITCAP	^[A-Z].+
CAPWORD	^[A-Z] [a-z]+$
ALLCAPS	^[A-Z]+$
CAPSMIX	^[A-z]*([A-Z] [a-z]|[a-z] [A-Z]) [A-z]*$
ALPHANUMMIX	^[A-z0-9]*([0-9] [A-z]|[A-z] [0-9]) [A-z0-9]*$
ALPHANUM	^[A-z]+ [0-9]+$
UPPERCHAR	^[A-Z]$
LOWERCHAR	^[b-z]$
SHORTNUM	^[0-9] [0-9]?$
INTEGER	^-[0-9]+$
REAL	^-?[0-9]\.[0-9]+$
ROMAN	^[IVX]+$
HASDASH	-
INITDASH	^-
ENDDASH	-$
PUNCTUATION	^[,.;:?!]$
QUOTE	^["'']$

**Table 7:** Numbers of subtypes and possible feature groups in each feature type

**Feature Type**	**Subtype**	**# of Possible Feature Groups**
Word	-	= 7
POS	-	= 7
Orthographical	17	× 17 = 119
Word shape	2	× 2 = 14
Affix	2	× 2 × 3 = 42
Word conjunction	-	= 21
Total	-	210

**Table 8:** The most effective word conjunction feature groups

**Feature groups**
*w*_*i*-1 _AND *w*_*i*_
*w*_*i*-2 _AND *w*_*i*-1_
*w*_*i *_AND *w*_*i*+1_
*w*_*i*-2 _AND *w*_*i*_
*w*_*i*-1 _AND *w*_*i*+1_
*w*_*i*-3 _AND *w*_*i*-1_
